# Nasal airway epithelial cell IL-6 and FKBP51 gene expression and steroid sensitivity in asthmatic children

**DOI:** 10.1371/journal.pone.0177051

**Published:** 2017-05-11

**Authors:** Michael Fayon, Aurelie Lacoste-Rodrigues, Pascal Barat, Jean-Christophe Helbling, Fabienne Nacka, Patrick Berger, Marie-Pierre Moisan, Jean-Benoit Corcuff

**Affiliations:** 1 Université de Bordeaux, Centre de Recherche Cardio-thoracique de Bordeaux, U1045, Bordeaux, France; 2 CHU de Bordeaux, Centre d’Investigation Clinique (CIC 1401), Bordeaux, France; 3 Université de Bordeaux, Nutrition and Integrative Neurobiology, Bordeaux, France; 4 INRA, UMR1286, Nutrition and Integrative Neurobiology, Bordeaux, France; National and Kapodistrian University of Athens, GREECE

## Abstract

**Background:**

Many asthmatic patients exhibit uncontrolled asthma despite high-dose inhaled corticosteroids (ICS). Airway epithelial cells (AEC) have distinct activation profiles that can influence ICS response.

**Objectives:**

A pilot study to identify gene expression markers of AEC dysfunction and markers of corticosteroid sensitivity in asthmatic and non-asthmatic control children, for comparison with published reports in adults.

**Methods:**

AEC were obtained by nasal brushings and primary submerged cultures, and incubated in control conditions or in the presence of 10 ng/ml TNFalpha, 10^-8^M dexamethasone, or both. RT-PCR-based expression of FKBP51 (a steroid hormone receptor signalling regulator), NF-kB, IL-6, LIF (an IL-6 family neurotrophic cytokine), serpinB2 (which inhibits plasminogen activation and promotes fibrin deposition) and porin (a marker of mitochondrial mass) were determined.

**Results:**

6 patients without asthma (median age 11yr; min-max: 7–13), 8 with controlled asthma (11yr, 7–13; median daily fluticasone dose = 100 μg), and 4 with uncontrolled asthma (12yr, 7–14; 1000 μg fluticasone daily) were included. Baseline expression of LIF mRNA was significantly increased in uncontrolled *vs* controlled asthmatic children. TNFalpha significantly increased LIF expression in uncontrolled asthma. A similar trend was observed regarding IL-6. Dexamethasone significantly upregulated FKBP51 expression in all groups but the response was blunted in asthmatic children. No significant upregulation was identified regarding NF-kB, serpinB2 and porin.

**Conclusion:**

LIF and FKBP51 expression in epithelial cells were the most interesting markers of AEC dysfunction/response to corticosteroid treatment.

## Introduction

Asthma is characterized by inflammation of the airways, reversible airflow obstruction and bronchial hyperresponsiveness. In many children, asthma is difficult to manage and remains poorly controlled, despite treatment with high-dose inhaled corticosteroids (ICS) (≥ 800 μg/d budesonide)[1–3]. Adverse home environments, poor treatment supervision, alternative diagnoses and unresponsiveness to corticosteroids (CS) are thought to be the most important influencing factors in difficult asthma [3]. The overall response to CS at a group level is heterogeneous and depends on the outcomes being studied [4]. Although rare cases of poor glucocorticoid receptor function have been described in children [2], the concept of variable corticoresponsiveness is more interesting to investigate than complete corticoresistance, which is rare [4]. Phenotyping patients according to underlying molecular characteristics may help in determining the prognosis and personalizing treatment regimens [5].

Resident lung cells, including airway epithelial cells (AEC) and smooth muscle cells, play an important role in effecting or perpetuating the airway response to T helper type 2 cytokines [6, 7]. AEC are now recognized as playing an important role in the inflammatory response to inhaled exposures in addition to the barrier effect which prevents the entry of inhaled matter into the lung parenchyma [7]. Recent evidence has shown that AEC from children with mild asthma are intrinsically different both biochemically and functionally compared with epithelial cells from children without asthma [8]. AEC from patients with asthma spontaneously produce significantly greater amounts of interleukin-6 (IL-6), prostaglandin E2 and epidermal growth factor, but significantly lower amounts of transforming growth factor 1 [8]. These abnormalities are evident early in the disease progression and correlate with disease severity.

CS may have direct effects on the epithelial cells themselves. In a genome-wide profiling study evaluating treatment response of adult asthmatic human AEC to CS, high baseline expression of CLCA1 (chloride channel, calcium-activated, family member 1), periostin, and serpinB2 (serine peptidase inhibitor, clade B (ovalbumin) member 2, a protease which inhibits plasminogen activation and promotes fibrin formation and deposition) were associated with a good clinical response to CS, whereas high expression of FKBP51 (FK506-binding protein 51, a regulator of steroid hormone receptor signalling) was associated with a poor response [6]. There is a lack of such studies in children. We hypothesized that asthmatic AEC molecular characteristics vary in an age-wise manner. Thus, similarly to a study in adults (6), our main aim was to identify gene expression markers of AEC dysfunction and markers of corticosteroid sensitivity in children. Based on Woodruff et al.’s study (6) and previous studies from our lab [9, 10], we decided to measure serpinB2 and FKBP51 as well as NF-kB, IL-6 [8], LIF (leukaemia inhibitory factor, an IL-6 family neurotrophic cytokine [9, 11]) and porin gene expression (a marker of mitochondrial mass [10, 12]) in AEC from children with no, controlled or uncontrolled asthma. The inflammatory and CS responses of these cells in each group of patients were studied by the addition of an inflammatory cytokine (TNFalpha) and/or dexamethasone [13].

To explore AEC dysfunction in childhood asthma and its association with the actions of CS, we collected epithelial cells from upper airways by using nasal brushings and examined expression profiles by RT-PCR.

## Material and methods

The present prospective pilot study was conducted at Bordeaux University Hospital, France, from May 2008 to December 2008. It was approved by Bordeaux’s Institutional Review Board on Human Research. All of the volunteers and their parents gave written informed consent.

### Patients

Nasal brushings were obtained from male and female asthmatic children aged < 18 years. Asthma was defined as physician-diagnosed asthma according to GINA criteria (http://www.ginasthma.org/). Controlled asthmatic patients had daytime asthma symptoms, night waking due to asthma, and required a reliever for symptoms less than twice per week, with no activity limitation due to asthma. Symptom control (GINA) had been obtained for at least 3 months prior to the study. The uncontrolled group presented with ≥ 3 of the above criteria, and most patients met modified ATS criteria (*i*.*e*. ≥ 800 μg/d equivalent dose of budesonide) [14]. Matched (age ± 1 yr, sex) control patients consisted of children undergoing elective surgery for non-respiratory conditions, without any history of asthma or allergy or recent upper airway infection, i.e. less than 1 month prior to inclusion. No patient had received topical nasal steroids 15 days prior to inclusion.

The following data were collected: age, sex, age upon diagnosis of asthma, atopic status as determined by a positive skin prick test (wheal diameter ≥ 3 mm) and/or RAST (>0.1 kUI/L) to common allergens, asthma control, lung function parameters and current treatment.

### Nasal brushings

Nasal brushings were performed either in the outpatient department, without topical anaesthesia, or under general anaesthesia in the operating theatre. The following brushes were used: 1-mm bronchoscope cytology brush, (Cook® Ireland Ltd.) or 3-mm urethral brush (Scrinet®, Laboratoire CCD, Paris—France). Nasal epithelial cells were harvested by gently brushing the inferior nasal turbinate [15].

### Cell isolation

Primary cultures of nasal AEC were grown according to the submerged culture model in Petri dishes coated with collagen as previously described [16] using dedicated epithelial culture medium [13] from Clonetics/Lonza*®* (Basel, Switzerland). All experiments were performed on phenotypically confirmed AEC between passages 1 and 2. In order to rule out any possible effect linked to the presence of hydrocortisone in the epithelial culture medium, AEC were placed in DMEM medium (Sigma, glucose 1g/L + antibiotics (penicillin, 100 000 units/L and streptomycin 100 mg/L) plus an anti-fungal agent (amphotericin 250μg/L) for 48 hours and then studied in control unstimulated conditions or in the presence of 10 ng/ml TNFalpha (4 hours) [17], 10^-8^M dexamethasone, or both [13, 18] (initial titration assays were performed using 10^−7^ and 10^-8^M dexamethasone; the latter was deemed a satisfactory concentration). RNA was extracted 4 hours post-stimulation.

### RNA extraction

Total RNA was extracted using a kit (TRIzol reagent, Invitrogen, France) according to the manufacturer ‘s protocol. Briefly, cells were lysed in the TRIzol buffer which contains phenol and guanidine isothiocyanate. Chloroform was then added and after centrifugation RNA was recovered in the aqueous phase and precipitated by addition of isopropyl alcohol. RNA was then resuspended in RNAse-free H_2_0 and RNA concentration was determined by spectrophotometry on a NanoDrop ND-1000 spectrophotometer (Labtech, France). RNA integrity was verified using the RNA-6000 NanoLabChip kit combined with a 2100 Bioanalyser (Agilent Technologies).

### Quantitative Polymerase Chain Reaction (RT-PCR)

First, total RNA (500 ng) was reverse-transcribed in cDNA with Superscript III (Invitrogen, Cergy Pontoise, France) and random hexamers according to the manufacturer’s protocol. Then 5 μl of the cDNA solution diluted 1:20 was added to 10μl of 2X concentrated buffer containing the Taq polymerase (LC480 SYBER-GREEN I Master solution, ROCHE) and 5μl of the target gene primers mix (300nM concentration) in a total volume of 20 μl. This mix was loaded onto a LightCycler^®^ 480II system (Roche) for PCR amplification of target genes. The PCR program consisted of 40 cycles of 95°C for 10s, 62°C for 6s and 72°C for 10s.

**Fig 1:**
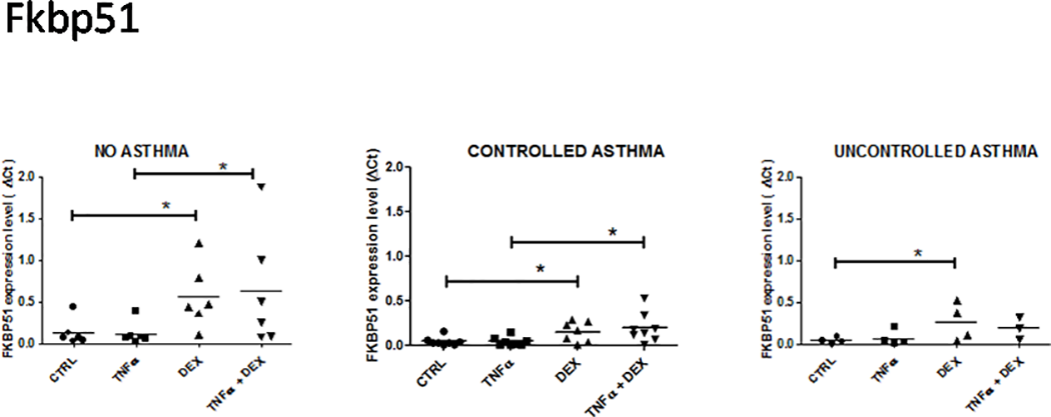
Expression of FK506-binding protein 51 (FKBP51) mRNA in control and asthmatic children in unstimulated and stimulated (TNFalpha + Dexamethasone) airway epithelial cells. Control patients, n = 8; controlled asthma, n = 6; uncontrolled asthma, n = 4. Results are means ± SEM. * *p* < 0.05.

**Fig 2:**
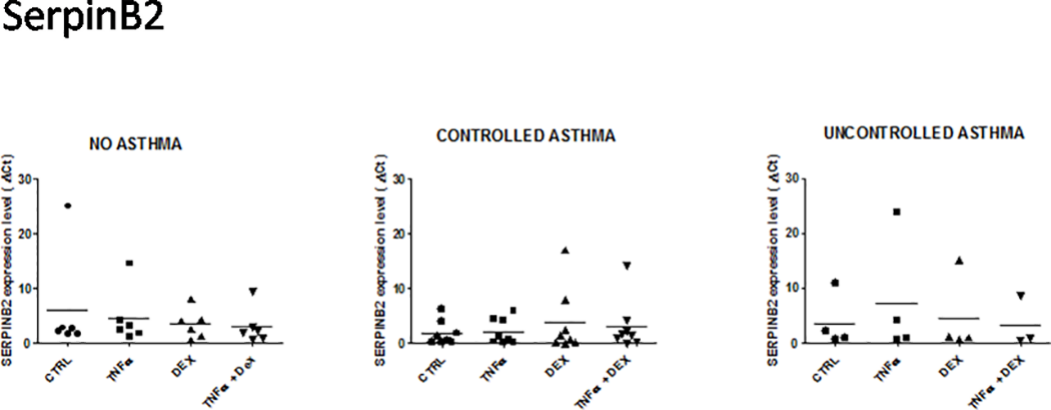
Expression of serine peptidase inhibitor, clade B (ovalbumin), member 2 (serpinB2) mRNA in control and asthmatic children in unstimulated and stimulated (TNFalpha ± dexamethasone) airway epithelial cells. See Fig 1 for legends.

**Fig 3:**
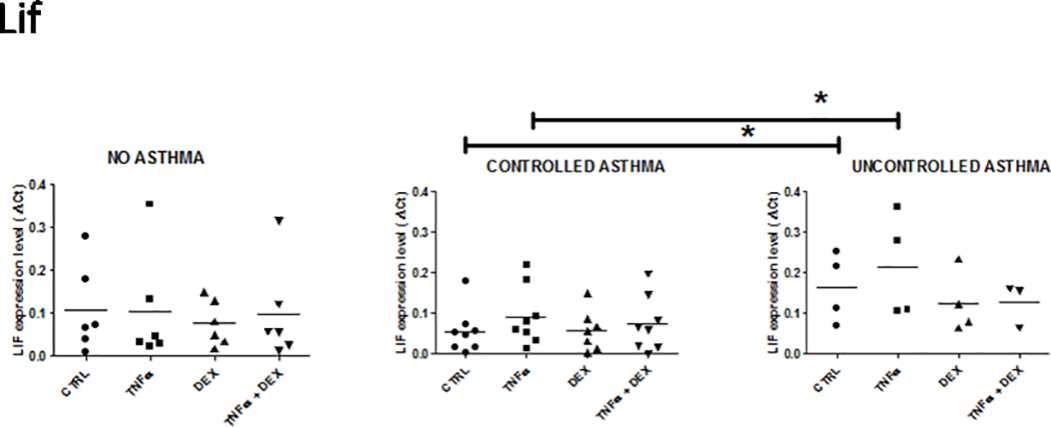
Expression of LIF mRNA in control and asthmatic children in unstimulated and stimulated (TNFalpha ± Dexamethasone) airway epithelial cells. See Fig 1 for legends.

**Fig 4:**
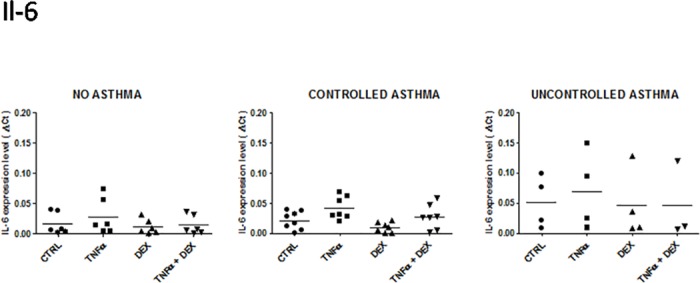
Expression of IL-6 mRNA in control and asthmatic children in unstimulated and stimulated (TNFalpha ± Dexamethasone) airway epithelial cells. See Fig 1 for legends.

**Fig 5:**
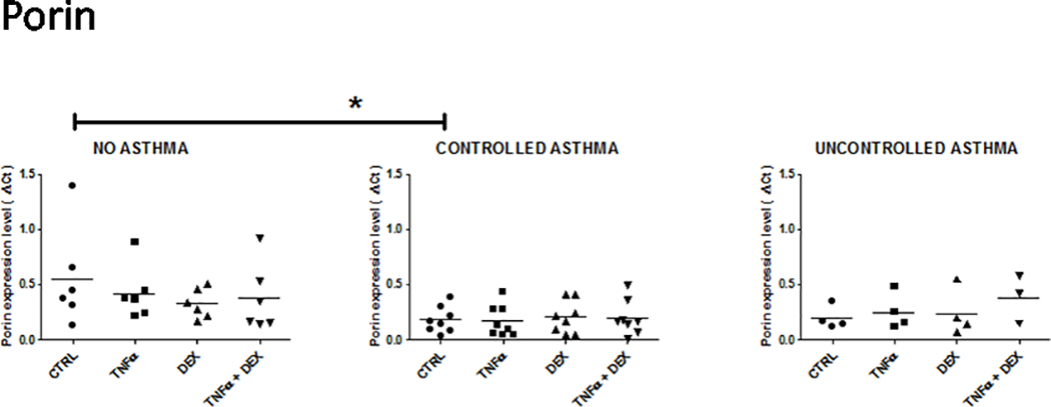
Expression of porin mRNA in control and asthmatic children in unstimulated and stimulated (TNFalpha ± dexamethasone) airway epithelial cells. See Fig 1 for legends.

**Fig 6:**
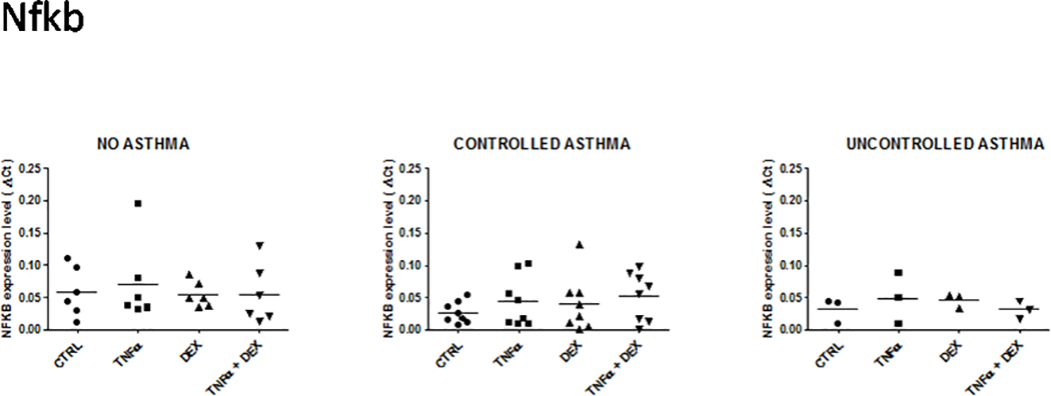
Expression of NF-kB mRNA in control and asthmatic children in unstimulated and stimulated (TNFalpha ± dexamethasone) airway epithelial cells. See Fig 1 for legends.

To avoid genomic DNA amplification, primer pairs were designed in two different exons (thus spanning an intron) using the Primer Express software (PE APPLIED Biosystems, Courtaboeuf, France). Sequences of primers used are provided in Table 1. The specificity of the PCR reaction was validated according to MIQE (Minimum Information for publication of Quantitative real time PCR Experiments) guidelines [19]. The mRNA levels of target genes were normalized using 18S RNA expression for each sample (cDNA diluted 1:2000). The 18S expression varied neither between stimulated and non-stimulated conditions nor among patients of the whole cohort. The quantification of target mRNA levels was calculated with the LightCycler480 Relative Quantification software (version 1.5). The expression values used in Figs 1–6 were calculated using the [deltaCt] corresponding to the equation: deltaCt = 2^-(target gene Ct–Reference gene Ct)^. PCR efficiency was checked to be at 100% for each pair of primers (Table 1).

**Table 1:** Sequences of primers used for Quantitative Polymerase Chain Reaction.

Protein	Primer
IL6-F2	GGTACATCCTCGACGGCATC
IL6-R3	GCCTCTTTGCTGCTTTCACAC
LIF-F2	GAACCAGATCAGGAGCCAACTG
LIF-R3	CCACATAGCTTGTCCAGGTTGTT
SERPINB2-F4	GTTATCCTGATGCGATTTTGCA
SERPINB2-R5	AGAGCGGAAGGATGAATGGA
Porin-F4	CAGAGAAATGGAATACCGACAATACA
Porin-R4-5	TCCACGTGCAAGCTGATCTT
NF-KB-F16	AAGTCACATCTGGTTTGATTTCTGAT
NF-KB-R16-17	AAGTGCAAGGGCGTCTGGTA

IL6: interleukin 6; LIF: leukaemia Inhibitory factor; SERPIN: serine peptidase inhibitor, clade B (ovalbumin) member 2; NF-KP: nuclear factor-kappa B.

### Statistical analysis

All values are median (min, max) or mean ± standard error of the mean (SEM). Statistical analysis was performed using the software package, NCSS 6.0.21, Kaysville, Utah, USA. Data were analysed for statistical significance using, as appropriate, the unpaired Student *t*-test, the Mann and Whitney U-test or a one-way analysis of variance (ANOVA). A *p* value < 0.05 was considered significant.

## Results

### Demographic data

Subject demographic data are provided in Table 2. The study population consisted in 6 control patients without asthma or lung disease (median age 11 yr, min-max: 7–13), 8 with controlled asthma (11 yr, 7–13), daily ICS dose (mean ± SD) 140 ±167 μg fluticasone (beclomethasone (eq.) dose (μg/day) 100 (0–400)) and 4 patients with uncontrolled asthma (12 yr, 7–14), daily ICS dose 560 ± 630 μg fluticasone (beclomethasone (eq.) dose (μg/day) 1000 (500–2000)).

**Table 2:** Demographic characteristics of patients.

	No Asthma	Controlled Asthma	Uncontrolled Asthma
n	6	8	4
Age (yr)	11 (7–13)	11 (7–13)	12 (7–14)
Male/Female (n/n)	4/2	6/2	2/2
Age at diagnosis of asthma (mo)		27 (3–108)	15 (11–24)
Asthma severity @ inclusion (GINA)		2 (1–2)	4 (4–4)
Atopy (n/N)		7/8	3/4
Passive smoking (n/N)		1/8	3/4
Beclomethasone (eq.) dose (μg/day)		100 (0–400)	1000 (500–2000)
LABA (n/N)		0/8	4/4
LRTA (n/N)		1/8	2/4
Omalizumab (n/N)		0	1/4
Baseline FEV_1_ (% predicted)		81 (68–117)	86 (70–100)
Baseline FEF_25-75_ (% predicted)		60 (43–112)	75 (42–98)

Median (min-max), FEV1: Forced expiratory volume in 1 second; LABA: Long-Acting Beta-Agonists; FEF25-75: forced expiratory flow at 25–75% of forced vital capacity; LRTA: Leukotriene-Receptor Antagonists

### Epithelial cell gene expression according to asthma control (S1 Table)

#### FK506-binding protein 51 (FKBP51)

Compared to medium alone, dexamethasone significantly upregulated FKBP51 expression in AEC from all groups of patients (deltaCt (mean ± SEM) 0.56 ± 0.16, 0.26 ± 0.11, 0.26 ± 0.11 in non- asthmatic, controlled and uncontrolled asthmatics patients, respectively; *p* < 0.05) (Fig 1). In terms of absolute value, there was a trend towards greater dexamethasone-induced FKBP51 expression in non-asthmatic patients than in controlled and uncontrolled asthmatics. TNFalpha alone did not induce any significant effect.

#### SerpinB2 (Serine peptidase inhibitor, clade B (ovalbumin), member 2)

No significant difference in the expression of serpin was noted, irrespective of group or cell culture conditions (Fig 2). A high deltaCt (23.80) outlier value was found in 1 patient with uncontrolled asthma after stimulation by TNFalpha compared to the three other patients (4.13, 0.81 and 0.58). All four patients were included in the present analysis.

#### Leukaemia inhibitory factor (LIF)

The baseline expression of LIF was significantly higher in cells from severe uncontrolled vs controlled asthmatics (deltaCt: 0.163 ± 0.04 vs 0.06 ± 0.02, respectively; p = 0.027) (Fig 3). The difference in LIF expression between uncontrolled (deltaCt: 0.21 ± 0.06) and controlled asthmatics (0.09 ± 0.03); p = 0.04) remained significant with TNFalpha stimulation of the cell cultures. In both groups, the decrease in LIF expression by dexamethasone in the presence of TNFalpha did not reach statistical significance (deltaCt, 0.12 ± 0.03 (uncontrolled) *vs* 0.07 ± 0.02 (controlled); NS). Overall, LIF levels in uncontrolled asthma were not different to those in controls owing to the presence of one outlier in the control group. Exclusion of this outlier led to a significant difference between the TNFalpha uncontrolled and non-asthmatic groups (deltaCt: 0.21 ± 0.06 (uncontrolled) *vs* 0.05 ± 0.02 (no-asthma); *p* = 0.033). This outlier may be due to variability in the control patients.

#### Interleukin 6 (IL-6)

Overall, the expression of IL-6 was similar to what was observed for LIF expression (Fig 4). There was a trend towards a higher baseline level of IL-6 in the uncontrolled asthmatic group in comparison to the non-asthmatic and non-asthmatic groups. However, no statistically significant difference was noted, irrespective of the group or cell culture conditions.

#### Porin

Porin gene expression appeared to be greater in non-asthmatic than in asthmatic patients (Fig 5). This result reached statistical significance in baseline conditions in AEC from patients with controlled asthma *vs* non-asthmatic patients only (deltaCt: 0.18 ± 0.04 *vs* 0.55 ± 0.18, respectively, *p* = 0.04).

#### Nuclear factor-kappa B, NF-kB

No significant difference was noted in NF-kB expression, irrespective of group or cell culture conditions (Fig 6).

## Discussion

The two main results of the present study involving cultured AEC from children are as follows. Firstly baseline and TNFalpha stimulation significantly increased the expression of LIF mRNA in children with uncontrolled asthma. Secondly, dexamethasone significantly upregulated FKBP51 expression but this response was blunted in children with uncontrolled asthma (i.e. with increased disease severity and inhaled steroid dose).

### Study rationale

Bronchial AECs remain the gold standard for asthma research [7, 17]. However, nasal AECs may be a suitable surrogate for the study of certain aspects of bronchial AEC function. For instance, in one study, a 90.2% overlap in expressed genes and a strong correlation in gene expression (ρ = 0.87) was found between the nasal and bronchial transcriptomes [20]. Similarly, within-subject correlation between nasal and bronchial production of transforming growth factor (TGF)-beta2 (r = 0.64, p = 0.001) and vascular endothelial growth factor (r = 0.73, P < 0.001) was good [21], although the release of IL-6, IL-8, and granulocyte colony-stimulating factor was reported to be significantly greater in nasal than in bronchial AEC [17].

A defective epithelial layer results in the exposure of the submucosa to a variety of environmental stimuli (allergens, microbes, pollutants), inducing sustained activation of the epithelial mesenchymal trophic unit [22]. Impaired barrier integrity and delayed repair [23, 24] and lower levels of TGF-beta1 [25] may be strongly linked to bronchial hyperresponsiveness. It is thus of utmost importance to understand fully the factors influencing AEC function and response to therapy.

The important role of atopy on the airway epithelium has already been shown in several studies [15, 20, 26, 27]. Allergic asthma and rhinitis remain complex diseases and more than 20 epithelium-derived biomarkers are currently available [26, 27]. In the future, these may be used alone or in combination as prognostic genes at different stages of the disease. In vitro, nasal AEC gene expression in children with house dust mite allergic rhinitis indicated a Th2-driven mechanism, highlighting the influence of epithelially-expressed molecules on asthma control, in association with altered responses to viruses [15, 24]. Similarly, nasal expression profiling has been proposed for identifying individuals with IL13-driven asthma and a Th2-skewed systemic immune response [20]. In some studies, however, atopy was not deemed to be an essential component in the disease process, and no differences in mediator release were noted between children with atopic asthma and those with virus-induced wheeze or between non-atopic and atopic controls [28].

The rationale of the present study was based on the hypothesis that age may have a marked influence on epithelial gene expression, given the age-related variations in asthma clinical phenotypes [29], inflammatory cascades in bronchial epithelial [30] and smooth muscle cells function [31]. The choice of the target genes and stimulating agents was based on data obtained from the literature (serpinB2 [6], FKBP51 [6] and from our own laboratory. First, the mechanisms leading to steroid-resistant asthma may involve enhanced expression of NF-kB and/or activator protein-1 [5]. Excessive active NF-kB, a critical transcription factor for the production of inflammatory cytokines, may reduce the anti-inflammatory properties of CS [32]. Second, LIF and the neurokinin receptor NK-1R are largely co-expressed in lung tissue in a rat asthma model [33]. LIF plays an important role as regulator of neurogenic inflammation [34], acting either as a pro- or anti-inflammatory cytokine, during an acute inflammatory insult and its resolution [11, 35]. In immature ASMC, LIF secretion enhances airway reactivity and [Ca^2+^]i signalling [9]. Increased LIF expression in airway epithelia of asthmatic rats is down-regulated by dexamethasone. Third, in vitro, the mass of asthmatic ASM mitochondria, as assessed by porin content, is increased as compared to that of non-asthmatics. Moreover, within the adult asthmatic population, both the duration of the disease and the FEV1/FVC ratio are correlated with porin content [10]. Fourth, TNFalpha is a pro-inflammatory cytokine that has been implicated in many aspects of the airway pathology in asthma and has previously been used to stimulate AEC [28]. Treatment of AEC with TNFalpha and IL-1βL-1 increased secretion of IL-6, IL-8, GSF, RANTES, MCP-1, VEGF, MMP-9, and TIMP-1[17]. Evidence suggests that it may play an important role in severe, refractory airway disease [36].

### Genes differentially expressed in paediatric asthmatic AEC

By using gene expression microarrays in adult asthmatic AEC, it was shown that serpinB2 is upregulated (3.5-fold in asthma vs controls) [6], unlike in the present study in children. In school-aged children, the allergic rhinitis /healthy nasal AEC serpinB2 log2 ratio was 1.64 [15]. In other studies, serpinB2 expression in bronchial AEC was modestly increased (1.491- and 1.871-fold change in asthmatic and atopic adolescents vs controls, respectively) [20].

In the present study, LIF, and to a lesser degree IL-6, expression was increased in asthmatic *vs* non-asthmatic children, with a further increase in uncontrolled asthma *vs* controlled asthma. The response was enhanced in the presence of TNFalpha, and LIF levels in uncontrolled asthma were also different to those of non-asthmatic patients when one outlier was omitted in the control group. This is similar to adults, in whom LIF is constitutively expressed in epithelial cells and is increased in the serum in mild asthmatics [11].

Adult AEC also produces an excess of inflammatory and pro-remodelling cytokines such as IL-6 [37]. In genome-wide association studies (GWAS) in adult asthmatics and controls, asthma risk significance was reached (OR 1**・**09, combined p = 2**・**4×10–⁸) for the IL-6 receptor gene [38]. The IL-6R coding SNP rs2228145 (Asp358Ala) is a potential modifier of lung function in subjects with asthma and may identify subjects at risk for more severe asthma [39]. However, there are inconsistencies between studies regarding AEC IL-6 release, which is not increased in many children with asthma vs controls [7].

The decrease in porin expression in children was unexpected given recent data indicating increased mitochondrial biogenesis and dysfunction [40] in asthmatic rat AEC [12], adult asthmatic AEC [41] and human ASM in culture [10, 42]. In asthmatic rat AEC, mitochondria are altered and swollen [43], with decreased mitochondrial basement membrane density and cristae [12]. Mitochondrial dysfunction and excessive production of reactive oxygen species promote allergic asthma and inhibition of Ca2+/calmodulin-dependent protein kinase II targeted to AEC mitochondria abrogates asthma [44].

### Cellular response to corticosteroids

The beneficial effects of corticosteroids in asthma could relate to their ability to decrease AEC activation by inflammatory cells and cytokines [6]. FKBP51 is considered to be a molecular marker of glucocorticosteroid response [45]. Long-term ICS in asthmatic adults markedly unregulated AEC FKBP51 and a higher expression of this molecule was associated with a poor response to ICS [6], in particular in the central airways [45]. Indeed, baseline FKBP51 expression correlated inversely with FEV1 response to fluticasone (at 8 weeks (*r* = -0.63, *p =* 0.009)). In our study in children, although dexamethasone increased FKBP51 expression, the absolute response was blunted in the presence of asthma. Differences compared to adults may be related to the epigenetic upregulation of the FKBP5 gene with increasing age, with environmental factors and airway inflammation acting as the epigenetic milieu [46]. Resistance to CS in patients with severe asthma may be an acquired process [47], possibly due to altered autoregulatory mechanisms.

In a clinical setting, long-term ICS also down-regulated the serpinB2 gene in adults [6]. In the present in vitro study in children, dexamethasone produced a similar non-significant down-regulating trend regarding serpinB2, LIF and IL-6 expression *vs* TNFalpha alone.

### Limitations of study

The findings of this pilot study are limited by the small number of patients included. In addition, asthmatic children were undergoing ICS therapy. However, the use of nasal brushings allows patients with severe asthma phenotypes to be investigated without discontinuing their long-term therapy, since nasal deposition is negligible with mouth-breathing devices used in older children [15]. Data interpretation is also difficult owing to the fact that only gene expression was explored and not protein expression.

## Conclusion

Nasal epithelial activation and steroid sensitivity profiles differ in paediatric asthma compared to adult asthma. In children, LIF proved to be the most interesting marker in uncontrolled asthma in terms of AEC mRNA expression. Response to corticosteroid treatment was also different since although dexamethasone increased FKBP51 expression in all groups, the absolute response was blunted in the presence of asthma.

## Supporting information

S1 Table Supporting table. (XLSX)

## Abbreviations

AEC: Airway epithelial cells
CS: Corticosteroids
ICS: Inhaled Corticosteroids
ASMC: Airway smooth muscle cell
FEV1/FVC: Forced expiratory volume in 1 sec/Forced vital capacity
